# Epidemiological characteristics of COVID-19 travel-associated cases in Vojvodina, Serbia, during 2020

**DOI:** 10.1371/journal.pone.0261840

**Published:** 2021-12-23

**Authors:** Tatjana Pustahija, Mioljub Ristić, Snežana Medić, Vladimir Vuković, Mirjana Štrbac, Smiljana Rajčević, Aleksandra Patić, Vladimir Petrović

**Affiliations:** 1 Institute of Public Health of Vojvodina, Novi Sad, Serbia; 2 Department of Epidemiology, Faculty of Medicine, University of Novi Sad, Novi Sad, Serbia; 3 Department of Microbiology with Parasitology and Immunology, Faculty of Medicine, University of Novi Sad, Novi Sad, Serbia; Faculty of Science, Ain Shams University (ASU), EGYPT

## Abstract

Coronavirus disease 2019 (COVID-19) is currently the biggest public health problem worldwide. Intensive international travel and tourism have greatly contributed to its rapid global spreading. This study is the first comprehensive analysis of the epidemiological characteristics and clinical outcomes of the travel-associated COVID-19 cases in Vojvodina, Serbia, from March 6 to December 31, 2020 and it concerns permanent residents of Serbia. A cross-sectional study was conducted using data from the dedicated surveillance database of the Institute of Public Health of Vojvodina. Overall, 650 travel-associated COVID-19 cases were recorded in Vojvodina during the observed period, mainly imported from Bosnia and Herzegovina, followed by Austria and Germany (N = 195, 30%; N = 79, 12.15% and N = 75, 11.54%, respectively). The majority of cases were in the working-age groups, 18–44 and 45–64 years (56.46% and 34.15%, respectively). Overall, 54 (8.31%) patients developed pneumonia. In comparison to patients without pneumonia, those with pneumonia were older (mean age = 55.39 years vs. 41.34 years, *p*<0.01) and had a higher percentage of comorbidities (57.41% vs. 16.61%). Men were more likely to develop pneumonia than women (OR = 2.22; 95% CI: 1.14–4.30, *p* = 0.02), as well as those in retired-age group (OR = 4.11; 95% CI: 2.0–8.46, p<0.01). Obesity (OR = 14.40; 95% CI: 3.8–54.6, *p*<0.01), diabetes (OR = 9.82; 95% CI: 3.15–30.61, *p*<0.01) and hypertension (OR = 7.99; 95% CI: 3.98–16.02, *p*<0.01) were the most prominent main comorbidities as predictors of pneumonia. Our results represent general epidemiological and clinical dynamics of COVID-19 disease in Vojvodina. Also, they provide evidence that the predictors of pneumonia were: increasing age, male sex, having underlying comorbidities, an increasing number of days from the return to laboratory confirmation of COVID-19 (OR = 1.08, 95% CI: 1.03–1.12, *p*<0.01), as well as an increasing number of days from symptoms onset to diagnosis (OR = 1.14, 95% CI: 1.07–1.21, *p*<0.01), while anosmia and ageusia were protective factors for developing it (OR = 0.31, 95% CI: 0.12–0.79, *p* = 0.01).

## Introduction

Coronavirus disease 2019 (COVID-19) caused by the novel severe acute respiratory syndrome coronavirus 2 (SARS-CoV-2) is currently the biggest public health problem around the globe. The clinical spectrum of COVID-19 ranges from asymptomatic to severe critical forms with a potential fatal outcome. Although mild forms of the disease account for the largest proportion of all cases (more than 80%) [1, 2], there is a high percentage of severe (14%) and critical (5%) clinical outcomes of COVID-19 with a high case fatality rate (CFR estimates by country—from less than 0.1% to over 25%) [1, 3]. During the ongoing pandemic, more than 174 million confirmed cases and 3.7 million deaths had been reported worldwide by June 12, 2021 [4].

Intensive international travel and tourism significantly contributed to the rapid spread of the virus from the city of Wuhan, Hubei province in China, where it was first registered in December 2019, to the global extent. Thus, travel-associated cases could lead to an increased risk of SARS-CoV-2 transmission during the early phase of the COVID-19 pandemic and in a situation when countries managed to reduce the number of cases to very small. Reintroduction of the virus can cause deterioration of the existing epidemiological situation. On the other hand, in countries with widespread community transmission of the SARS-CoV-2 virus, travel-associated cases probably have little impact on the dynamics of the outbreak [5].

The first case of COVID-19 in the Republic of Serbia (Serbia) was recorded on March 6, 2020 in a traveler returning from Hungary. Until June 12, 2021, a total of 714,753 COVID-19 cases had been registered in Serbia [6], including 159,651 case in the Autonomous Province of Vojvodina (Vojvodina). In February, 2020 Serbian authorities introduced measures such as airport screening and medical surveillance of travelers from countries with intensive transmission of the virus. All passengers in the international traffic, who had suspicious COVID-19 symptoms or developed them within 14 days upon entering the country, were obligatory tested by reverse transcriptase polymerase chain reaction (RT-PCR) assay. Confirmed cases of COVID-19 were immediately isolated and their close contacts were promptly and thoroughly traced and placed into home isolation for 28 days (it was later shortened to 14 days and is still in force) since the last contact with the confirmed case. Regular check-ups of symptoms were performed by epidemiologist, while the police monitored daily compliance with isolation of close contacts. Those contacts that developed any symptoms/signs related to COVID-19 were tested by RT-PCR. On March 15, a state of emergency was declared at the national level. Strict measures were implemented such as closing borders, movement restrictions, school, kindergartens and faculties closures, while all citizens aged 65 and above were recommended to stay indoors. For all returning travelers, mandatory home isolation was established for 14 to 28 days, depending on the epidemiological situation in the country they came from. The state of emergency lasted until May 6, 2020 and made a significant contribution to the control of the outbreak of COVID-19 in Serbia, nearly nullifying the first wave of the epidemic. Relaxation of measures with borders reopening and entry into the country without a mandatory RT-PCR test on 22 May, 2020, resulted, as expected, in the increase of the number of COVID-19 cases, leading to the second wave of outbreak. During the second (June, July) and third (November, December) wave of the COVID-19 outbreak in 2020, intensive community transmission was already underway. At that point, measures aimed at potential travel-associated COVID-19 cases were based on having a negative RT-PCR test result when entering the country. In the absence of this, home quarantine for 10 days was obligatory. This measure is still valid, but in addition to a negative RT-PCR test result, travelers can be exempted from home quarantine if they have evidence of complete vaccination against COVID-19 when entering the country [7].

The aim of this study was to summarize the epidemiological and clinical characteristics of travel-associated cases of COVID-19 in the Vojvodina, Serbia from March 6 to December 31, 2020.

## Materials and methods

### Study setting

We conducted a cross-sectional study. The study case was a resident of Vojvodina, who was laboratory-confirmed for the presence of the SARS-CoV-2 virus from March 6 until December 31, 2020 and who returned from a trip abroad within 14 days before the onset of symptoms/signs of COVID-19 or before the date of SARS-CoV-2 laboratory confirmation in case of the asymptomatic patients.

### Laboratory procedures

For the detection of SARS-CoV-2, patients’ posterior nasopharyngeal swab specimens were collected and tested with semi-quantitative RT-PCR. Depending on the availability of tests on the market, different ones were used: Liferiver Novel Coronavirus (2019-nCoV) Real-Time Multiplex RT-PCR kit, GeneFinder COVID-19 plus RealAmp kit, Biomerieux ARGENE® SARS-COV-2 R-GENE® Real-time detection kit and BGI Real-Time Fluorescent RT-PCR kit. The interpretation of the results was done in accordance with the manufacturer’s instructions [8–11]. Quantitative test COVID-19 Genesig Real-Time PCR Kit (Primerdesign Ltd, Chandler’s Ford, UK) was less commonly utilized and a detailed description of its usage and interpretation of the results has been previously published [12]. People in home isolation were tested on the site by a previously trained lab technician of the competent public health institutes. Later, this role was taken over by a lab technician of the competent health centers. After the introduction of antigen rapid diagnostic tests (Ag-RDT) into ambulatory practice in November 2020, patients in the early phases of the illness (5 days following symptom/sign onset) were tested using the STANDARD Q COVID-19 Ag Test (SD Biosensor, Gyeonggi-do, South Korea) [12]. Since a negative Ag-RDT result in the presence of COVID-19-like symptoms/signs cannot completely exclude an active COVID-19 infection, in order to definitive laboratory diagnose (positive or negative), the RT-PCR was performed [13]. Thus, a laboratory-confirmed COVID-19 case was defined as a person that resulted positive in an initial or repeated RT-PCR test or in an Ag-RDT for SARS-CoV-2.

### Data collection

The data originate from a specially constructed epidemiological questionnaire and were collected at the time of testing for the presence of the SARS-CoV-2 virus The questionnaire was comprised of questions about socio-demographic features, travel history in the past two weeks, date of return to Serbia, list of symptoms/signs and date of their onset, date of laboratory testing and COVID-19 status (positive/negative), main comorbidity, number of comorbidities and the hospitalization status. All collected data were entered into a dedicated surveillance database, created by the Institute of Public Health of Vojvodina, Novi Sad which covered all laboratory-confirmed COVID-19 patients in Vojvodina during the observed period.

Based on their place of residence patients were divided into seven administrative districts of Vojvodina: North, West, and South Bačka; North, Central, and South Banat; and Srem district.

Regarding the clinical presentation, travel-associated cases were classified into four laboratory-confirmed COVID-19 cases: 1) asymptomatic, if no symptom was present at the time of testing or during the epidemiological interview; 2) mild, if patients experienced any of the general infective, respiratory or digestive symptoms, without clinical or radiological confirmation of pneumonia; 3) severe, if COVID-19 pneumonia was confirmed and patients experienced any of the signs/symptoms related to COVID-19; and 4) critical disease was defined as COVID-19 pneumonia that required intubation, invasive mechanical ventilation and admission to the ICU. Based on their clinical status upon returning to Serbia, COVID-19 travel-associated cases were divided into two groups: symptomatic and asymptomatic upon return, and were analyzed accordingly. Data were extracted from the surveillance database and inappropriate outliers were assigned as missing for the subsequent analyses and statistical modeling.

### Statistical analysis

Descriptive analyses to summarize socio-demographic, epidemiological, and clinical characteristics of COVID-19 travel-associated cases overall, by the presence/absence of symptoms/signs upon return, employment category (working-age and retired-age), and by the presence of pneumonia, were performed. As quantitative data did not follow a normal distribution, in order to compare characteristics between different groups, the Mann-Whitney U test was used for continuous and discrete variables while Pearson’s chi-squared and Fisher’s exact test were used for the categorical variables.

Further, we used the logistic regression models to explore the predictive factors associated with pneumonia. Univariate analysis was conducted using independent variables: age; sex; number and the main type of comorbidities; days between symptoms onset and diagnosis, symptoms onset and date of hospitalization as well as days between return and the start of symptoms/signs onset and return and diagnosis; employment category (working-age or retired-age); presence of general, respiratory or digestive symptoms, and anosmia or ageusia. Based on the univariate analysis results and clinical significance of the explored variables, age, sex and the number of comorbidities were used for adjusting in the multivariate analyses. Odds ratios (OR) with the corresponding 95% confidence interval (95% CI) were calculated to express the strength of association. A p-value <0.05 was considered statistically significant across the analyses. All statistical analyses were performed using Stata, v.16 (STATA StataCorp, College Station, TX, USA) and maps were generated using the Quantum GIS (QGIS) software version 3.10.

### Ethical considerations

In accordance with applicable laws and regulations, no approval by the Ethics Committee for the retrospective analysis of anonymized data is required in Serbia. The authors of this study were not involved in the treatment of the patients that were included in the analysis, and data were anonymized before the authors accessed it.

## Results

**Fig 1:**
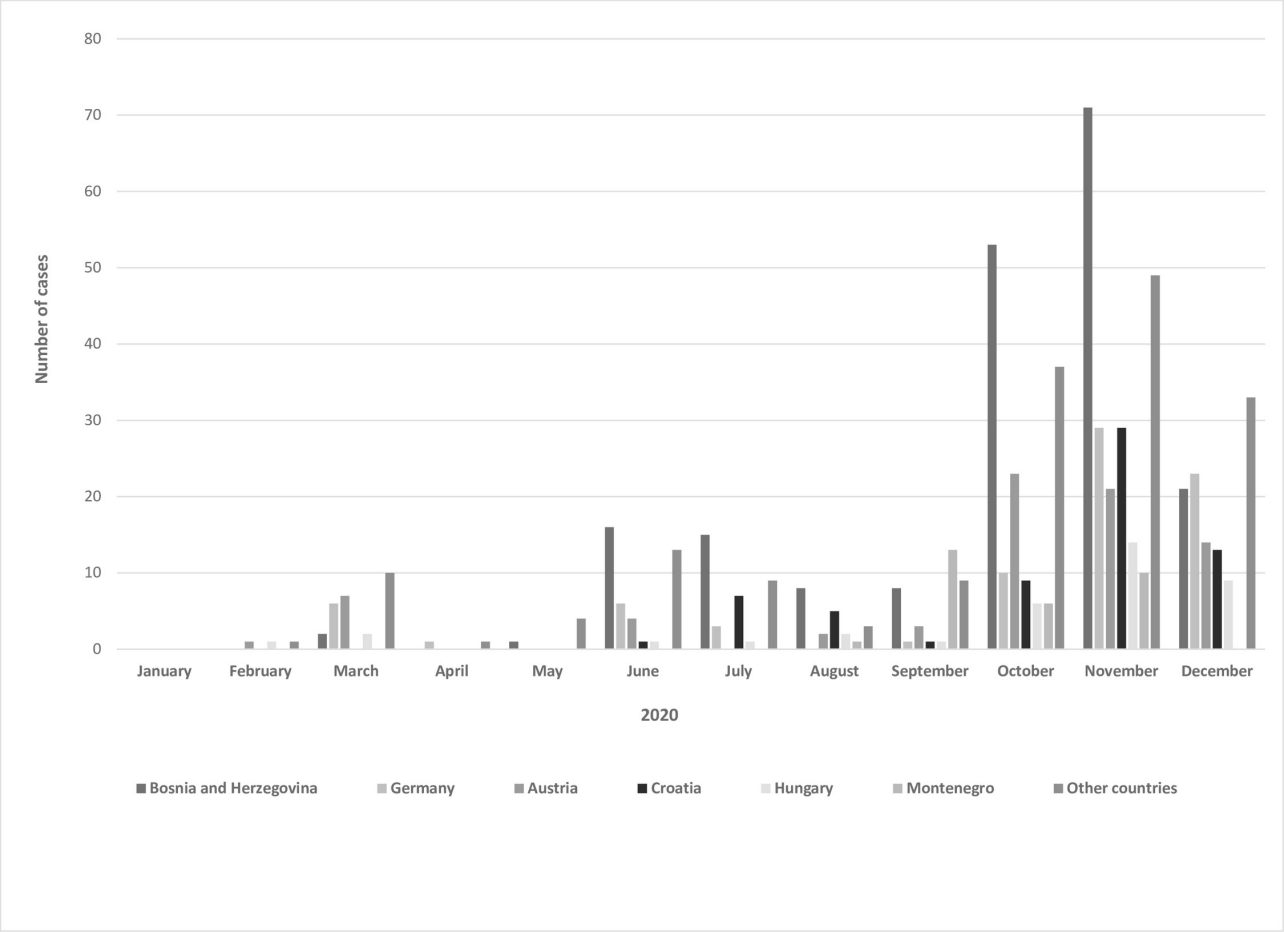
Monthly distribution of COVID-19 travel-associated cases in Vojvodina, Serbia, according to the date of entry and the country of importation.

Overall, 650 COVID-19 travel-associated cases were registered during the observed period in Vojvodina. The SARS-CoV-2 virus was imported to from 41 different countries around the world. The majority of travel-associated cases returned from Bosnia and Herzegovina (N = 195, 30%), followed by Austria and Germany (N = 79, 12.15%; and N = 75, 11.54%, respectively). One fifth of cases were imported from the neighboring countries (Croatia, Hungary and Montenegro) (S1 Table and Fig 1).

The distribution of the COVID-19 travel-associated cases by months of return and by countries of importation is shown in the Fig 1. Three waves of importation can be distinguished. Out of the total number of travel-associated cases, the lowest number (N = 37; 5.69%) was registered during the first wave of the outbreak in Vojvodina, while during the second and the third waves there were up to 133 (20.46%) and 480 (73.85%) cases, respectively.

Travel-associated cases were registered in all seven districts of Vojvodina with the highest incidence rate in South Bačka (68.25/100,000), followed by North Banat and West Bačka district (32.48/100,000 and 26.05/100,000, respectively).

Detailed socio-demographic, epidemiological and clinical characteristics of travel-associated cases are shown across Tables 1–4. The mean age of the patients was 42.51 years. The youngest travel-associated case was seven months, while the oldest one was 84 years old. The majority of cases were in the working-age groups. Males were found to be more prone to contracting COVID-19 (M/F ratio = 1.67:1). Every fifth travel-associated case had at least one comorbidity. Hypertension and diabetes were the most frequently reported as the main comorbidities. (Table 1).

**Table 1:** Sociodemographic, epidemiological and clinical characteristics of COVID-19 travel-associated cases, total and by clinical presentation upon return, in Vojvodina, Serbia, in the period March 6—December 31, 2020.

Variable	Total						Symptomatic upon return						Asymptomatic upon return						*p* value*
	N	mean	SD	median	IQR (25–75)		N	mean	SD	median	IQR (25–75)		N	mean	SD	median	IQR (25–75)		
**Age (years)**	650	42.51	14.37	41	31	53	526	43.86	14.21	43	32	54	124	36.78	13.61	36	26.5	44	**<0.01**
**Time between return and symptoms onset (days)**	526	5.12	4.10	4	2	8	526	5.12	4.1	4	2	8	NA	NA	NA	NA	NA	NA	NA
**Time between return and laboratory confirmation (days)**	650	7.74	5.75	7	3	12	526	8.52	5.84	8	4	13	124	4.47	3.94	3	1	7	**<0.01**
**Time between start of symptoms onset and laboratory confirmation (days)**	526	4.91	4.09	4	2	7	526	4.91	4.09	4	2	7	NA	NA	NA	NA	NA	NA	NA
**Time between symptoms onset and hospitalization (days)**	42	8.07	4.95	8	5	10	42	8.07	4.95	8	5	10	NA	NA	NA	NA	NA	NA	NA
**Sex male, n (%)**	407 (62.62)						326 (61.98)						81 (65.32)						0.49
**Age (years), n (%)**																			**<0.01**
<18	11 (1.69)						8 (6.45)						3 (0.57)						
18–44	367 (56.46)						86 (69.35)						281 (53.42)						
45–64	222 (34.15)						27 (21.77)						195 (37.07)						
65+	50 (7.69)						3 (2.42)						47 (8.94)						
**Comorbidity number, n (%)**																			
0	520 (80.00)						403 (76.62)						117 (94.35)						
1	103 (15.85)						97 (18.44)						6 (4.84)						
2	21 (3.23)						20 (3.80)						1 (0.81)						
≥3	6 (0.92)						6 (1.14)						0 (0)						
**Type of main comorbidity, n (%)**																			0.41
Obesity	10 (7.69)						9 (7.32)						1 (14.29)						
Diabetes	16 (12.31)						14 (11.38)						2 (28.57)						
Hypertension	63 (48.46)						61 (49.59)						2 (28.57)						
Malignant disease	2 (1.54)						2 (1.63)						0 (0)						
Cardiovascular disease	12 (9.23)						11 (8.94)						1 (14.29)						
Chronic lung disease	12 (9.23)						12 (9.76)						0 (0)						
Other chronic disease or condition	15 (11.54)						14 (11.38)						1 (14.29)						
**Type of COVID-19 test, n (%)**																			**<0.01**
RT-PCR	505 (77.69)						386 (73.38)						119 (95.97)						
Antigen rapid detection test	145 (22.31)						140 (26.62)						5 (4.03)						

*Indicators of significance between groups using Pearson’s chi-squared test and Fisher’s exact test for categorical and Wilcoxon rank-sum test for nonparametric continuous and discrete variables. Significance levels are given in bold for *p*<0.05. NA = not applicable.

**Table 2:** Comparison of sociodemographic and epidemiological characteristics of COVID-19 travel-associated cases between working and retired-age patients in Vojvodina, Serbia, in the period March 6—December 31, 2020.

Variable	Working-age imported cases (18–65 yrs.old)						Retired-age imported cases (65+)						*p* value*
	N	mean	SD	median	IQR (25–75)		N	mean	SD	median	IQR (25–75)		
**Age (years)**	589	40.77	11.75	40	31	50	50	70.04	4.72	69	67	72	**<0.01**
**Time between return and symptoms onset (days)**	476	5.22	4.12	4	2	8	47	4.30	3.80	4	1	6	0.15
**Time between return and laboratory confirmation (days)**	589	7.58	5.53	7	3	12	50	8.82	6.32	9	3	12	0.22
**Time between symptoms onset and laboratory confirmation (days)**	476	4.61	3.60	4	2	6	47	6.91	4.02	7	4	9	**<0.01**
**Time between symptoms onset and hospitalization (days)**	34	7.65	4.29	7	5	9	8	9.88	7.20	9,5	6	12	0.26
**Sex male, n (%)**	373 (63.33)						28 (56)						0.30
**Age (years), n (%)**													NA
<18	NA						NA						
18–44	367 (62.31)						NA						
45–64	222 (37.69)						NA						
65+	NA						50 (100)						
**Comorbidity number, n (%)**													**<0.01**
0	485 (82.34)						24 (48)						
1	85 (14.43)						18 (36)						
2	15 (2.55)						6 (12)						
≥3	4 (0.68)						2 (4)						
**Type of main comorbidity, n (%)**													0.34
Obesity	9 (8.65)						1 (3.85)						
Diabetes	11 (10.58)						5 (19.23)						
Hypertension	50 (48.08)						13 (50)						
Malignant disease	2 (1.92)						0 (0)						
Cardiovascular disease	8 (7.69)						4 (15.38)						
Chronic lung disease	12 (11.54)						0 (0)						
Other chronic disease or condition	12 (11.54)						3 (11.54)						
**Type of COVID-19 test, n (%)**													0.82
RT-PCR	456 (77.42)						38 (76)						
Antigen rapid detection test	133 (22.58)						12 (24)						
**Reported symptoms, n (%)**													
General infective symptoms	428 (72.67)						46 (92)						**<0.01**
Respiratory symptoms	345 (58.57)						29 (58)						0.94
Digestive symptoms	80 (13.58)						15 (30)						**<0.01**
Loss of smell or taste	149 (25.30)						4 (8)						**<0.01**
Fever	339 (57.56)						37 (74)						**0.02**
Cough	245 (41.60)						21 (42)						0.96
Sore throat	125 (21.22)						6 (12)						0.12
Weakness	268 (45.50)						31 (62)						**0.03**
Chills	4 (0.68)						0 (0)						0.56
Runny nose	132 (22.41)						7 (14)						0.17
Anosmia	135 (22.92)						4 (8)						**0.01**
Ageusia	105 (17.83)						3 (6)						**0.03**
Myalgia	119 (20.20)						20 (40)						**<0.01**
Arthralgia	74 (12.56)						10 (20)						0.14
Diarrhea	56 (9.51)						11 (22)						**<0.01**
Nausea	29 (4.92)						6 (12)						**0.04**
Vomiting	9 (1.53)						6 (12)						**<0.01**
Headache	180 (30.56)						13 (26)						0.50
Anxiety	19 (3.23)						2 (4)						0.77
Dyspnea	28 (4.75)						3 (6)						0.69
**Clinical presentation of COVID-19, n (%)**													**<0.01**
Asymptomatic	113 (19.19)						3 (6)						
Mild	434 (73.68)						35 (70)						
Severe	38 (6.45)						9 (18)						
Critical	4 (0.68)						3 (6)						
**Pneumonia, n (%)**	42 (7.13)						12 (24)						**<0.01**
**Hospital admission, n (%)**	34 (5.77)						8 (16)						**<0.01**

*Indicators of significance between groups using Pearson’s chi-squared test and Fisher’s exact test for categorical and Wilcoxon rank-sum test for nonparametric continuous and discrete variables. Significance levels are given in bold for *p*<0.05. NA = not applicable.

**Table 3:** Sociodemographic, epidemiological, and clinical characteristics of COVID-19 travel associated cases in Vojvodina, Serbia, in the period March 6—December 31, 2020, by pneumonia status.

Variable	Pneumonia (severe and critical)						Without pneumonia (mild and asymptomatic)						*p* value*
	N	mean	SD	median	IQR (25–75)		N	mean	SD	median	IQR (25–75)		
**Age (years)**	54	55.39	11.69	57	49	63	596	41.34	14.02	40	30.5	51	**<0.01**
**Time between return and symptoms onset (days)**	54	5.00	3.88	5	2	7	472	5.14	4.13	4	2	8	0.99
**Time between return and laboratory confirmation (days)**	54	10.33	6.45	10	5	15	596	7.51	5.63	7	3	11	**<0.01**
**Time between symptoms onset and laboratory confirmation (days)**	54	7.63	4.33	7	4	10	472	4.60	3.95	4	2	6	**<0.01**
**Time between symptoms onset and hospitalization (days)**	31	8.29	5.34	8	5	10	11	7.45	3.78	7	4	11	0.83
**Sex male, n (%)**	42 (77.78)						365 (61.24)						**0.02**
**Age (years), n (%)**													**<0.01**
<18	0 (0)						11 (1.85)						
18–44	9 (16.67)						358 (60.07)						
45–64	33 (61.11)						189 (31.71)						
65+	12 (22.22)						38 (6.38)						
**Comorbidity number, n (%)**													**<0.01**
0	23 (42.60)						497 (83.39)						
1	20 (37.04)						83 (13.93)						
2	7 (12.96)						14 (2.35)						
≥3	4 (7.41)						2 (0.34)						
**Type of main comorbidity, n (%)**													0.24
Obesity	4 (12.90)						6 (6.06)						
Diabetes	5 (16.13)						11 (11.11)						
Hypertension	17 (54.84)						46 (46.46)						
Malignant disease	0 (0)						2 (2.02)						
Cardiovascular disease	3 (9.68)						9 (9.09)						
Chronic lung disease	0 (0)						12 (12.12)						
Other chronic disease or condition	2 (6.45)						13 (13.13)						
**Type of COVID-19 test, n (%)**													**<0.01**
RT-PCR	50 (92.59)						455 (76.34)						
Antigen rapid detection test	4 (7.41)						141 (23.66)						
**Reported symptoms, n (%)**													** **
General infective symptoms	53 (98.15)						424 (71.14)						**<0.01**
Respiratory symptoms	44 (81.48)						331 (55.54)						**<0.01**
Digestive symptoms	12 (22.22)						83 (13.93)						0.10
Loss of smell or taste	5 (9.26)						148 (24.83)						**0.01**
Fever	50 (92.59)						328 (55.03)						**<0.01**
Cough	36 (66.67)						231 (38.76)						**<0.01**
Sore throat	7 (12.96)						125 (20.97)						0.16
Weakness	42 (77.78)						259 (43.46)						**<0.01**
Chills	0 (0)						4 (0.67)						0.55
Runny nose	5 (9.26)						135 (22.65)						**0.02**
Anosmia	2 (3.70)						137 (22.99)						**<0.01**
Ageusia	4 (7.41)						104 (17.45)						0.06
Myalgia	19 (35.19)						120 (20.13)						**0.01**
Arthralgia	9 (16.67)						75 (12.58)						0.39
Diarrhea	9 (16.67)						58 (9.73)						0.11
Nausea	7 (12.96)						28 (4.70)						**0.01**
Vomiting	5 (9.26)						10 (1.68)						**<0.01**
Headache	13 (24.07)						181 (30.37)						0.33
Anxiety	5 (9.26)						16 (2.68)						**<0.01**
Dyspnea	11 (20.37)						20 (3.36)						**<0.01**
**Clinical presentation of COVID-19, n (%)**													NA
Asymptomatic	0 (0)						124 (20.81)						
Mild	0 (0)						472 (79.19)						
Severe	47 (87.04)						0 (0)						
Critical	7 (12.96)						0 (0)						
**Hospital admission, n (%)**	31 (57.41)						11 (1.85)						**<0.01**

*Indicators of significance between groups using Pearson’s chi-squared test and Fisher’s exact test for categorical and Wilcoxon rank-sum test for nonparametric continuous and discrete variables. Significance levels are given in bold for *p*<0.05. NA = not applicable.

**Table 4:** Predictors of pneumonia (severe and critical disease) in travel-associated cases of COVID-19 in Vojvodina, Serbia, in the period March 6—December 31, 2020.

Odds of pneumonia	N	Unadjusted Model			N	Adjusted Model 1			N	Adjusted Model 2		
		OR	95% Conf. Interval	p-value*		OR	95% Conf. Interval	p-value*		OR	95% Conf. Interval	p-value*
**Age (years)**	650	1.07	1.05–1.10	**<0.01**	-	-	-	-	-	-	-	-
**Sex (male versus female)**	650	2.22	1.14–4.30	**0.02**	-	-	-	-	-	-	-	-
**Employment status (retired-age vs working age)**	639	4.11	2.00–8.46	**<0.01**	639	0.54	0.20–1.47	0.23	-	-	-	-
**Comorbidity number**												
0	650	**ref.**			650	**ref.**			-			
1		5.21	2.74–9.90	**<0.01**		3.03	1.52–6.03	**<0.01**	-	-	-	-
2		10.80	3.98–29.34	**<0.01**		4.79	1.66–13.83	**<0.01**	-	-	-	-
≥3		43.22	7.52–248.23	**<0.01**		28.28	4.40–181.74	**<0.01**	-	-	-	-
**Type of main comorbidity**												
No comorbidity	636	ref.			636	ref.			-			
Obesity		14.41	3.80–54.60	**<0.01**		10.87	2.64–44.75	**<0.01**	-	-	-	-
Diabetes		9.82	3.15–30.61	**<0.01**		4.72	1.44–15.51	**0.01**	-	-	-	-
Hypertension		7.99	3.98–16.02	**<0.01**		3.89	1.83–8.25	**<0.01**	-	-	-	-
Malignant disease		-	-	**-**		-	-	-	-	-	-	-
Cardiovascular disease		7.20	1.83–28.40	**<0.01**		3.27	0.74–14.49	0.12	-	-		-
Chronic lung disease		-	-	**-**		-	-	-	-	-	-	-
Other chronic disease or condition		3.32	0.71–15.60	0.13		2.58	0.51–13.03	0.25	-	-	-	-
Time between symptoms onset and laboratory confirmation (days)	526	1.14	1.07–1.21	**<0.01**	526	1.13	1.07–1.20	**<0.01**	526	1.14	1.07–1.20	**<0.01**
Time between symptoms onset and hospitalization (days)	42	1.04	0.89–1.20	0.63	-	-	-	-	-	-	-	-
Time between return and symptoms onset (days)	526	0.99	0.93–1.06	0.82	-	-	-	-	-	-	-	-
Time between return and laboratory confirmation (days)	650	1.08	1.03–1.12	**<0.01**	650	1.07	1.02–1.12	**<0.01**	650	1.07	1.02–1.12	**<0.01**
**Reported symptoms**												
General infective symptoms	650	21.5	2.95–156.70	**<0.01**	650	14.7	1.99–108.58	**<0.01**	650	12.09	1.62–90.02	**0.02**
Respiratory symptoms	650	3.52	1.74–7.13	**<0.01**	650	3.72	1.77–7.79	**<0.01**	650	3.48	1.63–7.41	**<0.01**
Digestive symptoms	650	1.77	0.89–3.49	0.10	-	-	-	-	-	-	-	-
Loss of smell or taste	650	0.31	0.12–0.79	**0.01**	650	0.49	0.19–1.30	0.15	650	0.40	0.15–1.09	0.07

*Significance levels are given in bold for *p*<0.05. Model1: adjusted for age (continuous) and sex. Model2: adjusted for age (continuous), sex and comorbidity number.

There were 526 (80.92%) patients with symptoms/signs related to COVID-19, with the average time between the onset of symptoms/signs and return to Vojvodina of 5.12 days. Among the symptomatic cases, the majority (70.15%; 369/526) developed symptoms 1 to 14 days (median 5 days, IQR = 3–9) before entering the country, 9.51% (50/526) on the date of arrival and in 20.34% (107/526) of patients the disease started 1 to 14 days (median 3 days, IQR = 2–5) after entering the country (S1 Fig).

The distribution of travel-associated cases by sex demonstrates that a mild form of COVID-19 prevailed in both sexes with a frequency of 69.77% (N = 284) in males and 77.36% (N = 188) in females. Among female travel-associated cases, no critical forms of COVID-19 were registered during the observed period (S2 Fig).

There were 589 (90.62%) travel-associated cases in the working-age group with the mean age of 40.77 years, and 50 (7.69%) retired-age travel-associated cases with the mean age of 70 years. The majority of patients, in both observed groups, were males. The time period between the symptoms onset and laboratory confirmation of the disease was significantly longer in the older age group. These patients more frequently had comorbidities (52%), severe and critical presentation of COVID-19 and they were admitted to the hospital more often, in comparison with the working-age group. On the other hand, asymptomatic and mild clinical forms of the disease as well as anosmia and ageusia were more frequently registered in the working-age group. The most frequent main comorbidities were hypertension, chronic lung disease and diabetes in the working-age group, while in the retired-age were hypertension, diabetes and cardiovascular disease (Table 2).

Socio-demographic, epidemiological and clinical characteristics of travel-associated cases with and without pneumonia, are presented in Table 3. Out of total cases, there were 54 (8.31%) patients that developed pneumonia and 31 (57.41%) of them were hospitalized. In comparison with patients without pneumonia, there were more males than females among patients with pneumonia and those patients were older compared to those without pneumonia. Patients who developed pneumonia had comorbidities in a higher percentage than those without pneumonia (57.41% vs. 16.61%), in particular those with two and ≥3 comorbidities. Patients with pneumonia had longer average time period between the return and the date of laboratory confirmation and between symptoms onset and laboratory confirmation. In comparison with patients without pneumonia, cases with pneumonia more frequently had general infective and respiratory symptoms, but less frequently had loss of smell or taste.

In the univariate analyses, increasing age was a predictor of developing pneumonia Men were more likely to develop pneumonia than women, as well as those in the retired-age group. The number of comorbidities was a strong predictor for the developing of pneumonia across all age categories, and the obesity, diabetes) and hypertension were the most prominent main comorbidities. In addition, an increasing number of days from the return to laboratory confirmation, as well as an increasing number of days from symptoms onset to laboratory confirmation of COVID-19 increased the probability of developing pneumonia among travel-associated cases. Cases with general infective as well as respiratory symptoms had the highest probability of developing pneumonia. Interestingly, loss of smell or taste was a protective factor, decreasing the odds of developing pneumonia. When adjusting the analyses for the effects of age and sex (model 1), similar results to those from the univariate analyses were obtained, with slightly weaker effects. After further adding the number of comorbidities as a covariate in model 2, the statistical significance of effects was confirmed and the results remained stable, across the explored variables (Table 4).

## Discussion

Previous experience of most countries showed that travel-associated cases were particularly important at the beginning of the COVID-19 pandemic when they significantly contributed to the local spread of the SARS-CoV-2 virus in importing countries, initiating local transmission by the appearance of clusters of cases in families and collectives, and then triggering outbreak wave [5, 14–18]. The extent of their impact gradually decreased over time, as the local incidence of cases increased. This certainly does not diminish the significance of analyzing their epidemiological and clinical characteristics, which may further contribute to better knowledge relevant to the epidemiology of this disease. Also, the analysis of epidemiological characteristics of travel-associated cases might be important to account for, when predicting the course of the epidemic in each country individually and is valuable for planning, introducing or adapting preventive measures against COVID-19.

To the best of our knowledge, this is the first study in this part of Southeast Europe that provided a detailed analysis of both socio-demographic and clinical characteristics of travel-associated cases and the predictors of pneumonia among them. Although there was included a relatively small sample of travel-associated cases, our results are comparable with the results obtained by studies that analysed laboratory-confirmed cases from the general population [2, 19–25]. Thus, we found that the majority of the confirmed cases had a mild clinical form of the disease, followed by asymptomatic. The most frequently reported symptoms or signs of COVID-19 were fever, weakness and cough. The disease was more common in the working-age group of patients. Men were more likely to contract COVID-19 and develop more severe forms of the disease and, therefore, they were more often hospitalized compared to women. Overall, the most common comorbidity was hypertension, followed by diabetes.

From March 6, 2020 to December 31, 2020, a portion of 0.83% (650/78,106) of all reported COVID-19 cases in Vojvodina was classified as travel-associated. Almost a third of these cases came from neighboring Bosnia and Herzegovina. This is not surprising, taking into account that a significant percentage of people living in Vojvodina originated from this country, so family visits and business connections were the most probable reason for the frequent travel to this country. Also, a large percentage of travel-associated cases was registered after returning from Austria and Germany. Although these countries are not adjacent to the territory of Serbia, a significant number of our citizens are temporarily living and working in these countries, mostly in service activities, and usually spend holidays in the native country. Furthermore, the importation of SARS-CoV-2 from Hungary is explained by trade connections with this country, with Montenegro by family ties and summer holidays on the coast and the reason for importing the virus from Croatia was mainly sea tourism. The topographic distribution of COVID-19 across the Vojvodina reveals the highest incidence rate in the South Bačka District. This can be explained by the fact that the population density in this district is the highest in Vojvodina and, at the same time, the main administrative center of Vojvodina (Novi Sad) is situated on its territory, so the odds for tourist travel of these citizens are higher and the health system is more accessible to them.

Our results revealed the evidence that mild clinical forms of COVID-19 disease were predominant in the total number of travel-associated cases in both sexes, which is consistent with the findings of other authors [26, 27]. It is a known fact that the proportion of asymptomatic infection of SARS-CoV-2 among travel-associated cases ranged from 3.4% in Taiwan [28] to 30.8% in Japanese citizens who were evacuated from Wuhan [29]. The results of our study showed that almost every fifth travel-associated case was asymptomatic. Similar to the findings obtained from other authors [30–32], we also found that men were more prevalent among the travel-associated cases, probably because they more often temporarily work abroad. Although both sexes have the same susceptibility to infection with SARS-CoV-2 virus, it was hypothesized that males are less likely to seek medical assistance for the same condition than women and possibly visit a doctor later with a more severe clinical status [33]. In addition, sex-based immunological differences and higher production of inflammatory cytokines, social and lifestyle factors, as well as behavioral patterns and the fact that men are more likely to have higher number of comorbidities may also be other potential explanations for more severe infection in men [31, 34, 35]. The majority of the travel-associated cases in our study were from the working age groups, 18–44 and 45–64, which was similar to the results obtained by the study encompassing early travel-associated COVID-19 cases in more than 40 countries [36]. Furthermore, comparing this age group and retired-age population, we found that working-age group was 11.8 times more likely to suffer from COVID-19. This can be explained by the fact that working age group travels more frequently, works abroad and makes business and private contacts much more often than the older age group. This group of people in our sample was three times less likely to have comorbidities than the retired-age group, thus explaining the higher proportion of mild and asymptomatic forms of the disease among them.

Regarding the differences between patients with and without pneumonia, we observed that the majority of travel-associated COVID-19 cases with pneumonia were males and belonged to the older age group. Results of the brief-review of the risk factors for COVID-19 severity previously concluded that the patient age and number of comorbidities are the most important risk factors [37]. Also, several explanations have been proposed for the possible association of older age and a higher risk for developing a severe clinical presentation of COVID-19. The existence of one or more underlying conditions in the elderly and also, possible repeated contacts with other infective agents throughout the life, might have caused antibody-dependent expansion and increasing inflammatory responses after contact with the SARS-CoV-2 virus, which consequently leads to a fulminant progression of the disease [38, 39]. We revealed the evidence that patients who developed pneumonia had a higher prevalence of comorbidities, and the number of comorbidities was a strong predictor for the development of pneumonia across all categories, similar to previous reports [40, 41]. In particular, obesity, diabetes and hypertension were the main comorbidities with the highest odds of developing pneumonia among study patients. Diabetes was previously described as one of the most critical comorbidities in respect to COVID-19 severity, potentially due to immunosuppressive effects caused by hyperglycemia [37]. Additionally, we found that the mean time from the arrival to laboratory confirmation of the disease was 7.74 days, which is consistent with the finding of the study that included early travel-associated cases from 144 countries [36]. This supports the epidemiological measure of quarantine for returnee travelers, as underlined before [42]. Longer time from the arrival to disease confirmation was the consequence of an asymptomatic disease or non-specific or very mild symptoms at the beginning of the illness, due to which the patients did not seek for medical help. It is a known fact that patients with suspicion of COVID-19 rarely consult a doctor if they have mild clinical form of the disease. Our results showed that the longer time period from the return to our territory to laboratory confirmation of the disease, as well as from the symptoms onset to laboratory confirmation of COVID-19, increases the probability of developing pneumonia. Extension of these time periods by each day increased the odds of developing pneumonia by 8% and 14%, respectively. We hypothesize this might be driven by higher percentage of males with pneumonia, who, as mentioned above, delay a medical consultation until the development of a more severe form of the disease [33].

Regarding symptomatic cases, even more than two-thirds of cases developed symptoms before returning to the country, with a median of 5 days, while in a study conducted in Taiwan half of the cases developed symptoms before arrival [28]. It remains unclear why patients traveled with COVID-19 symptoms. One of the explanations could be that they returned to Vojvodina because they were afraid of developing a more severe form of the disease, which could lead to hospitalization away from the family, and additionally many of those patients did not have medical insurance abroad. A further explanation might be that they returned to home country after the cessation of COVID-19 symptoms, but were still positive on the RT-PCR test, which they underwent after entering the country to be exempted from the mandatory home isolation. In our analyses, symptoms such as anosmia or ageusia, were demonstrated to be protective, i.e. showing lower odds of developing pneumonia, which might be due to the fact that these symptoms were more frequently reported by younger individuals and by women, and are mostly associated with mild clinical course of the disease [41, 43].

This study has some limitations that should be taken into account when interpreting our results. First, national testing protocols were restrictive and testing capacities were limited, especially at the beginning of the pandemic (March-April, 2020), thus the reported COVID-19 incidence rates may be underestimated. Second, although we had organized training among health care providers with the focus on the way and the importance of adequate data collection from COVID-19 patients, it is possible that the questionnaires were incomplete, especially when the presence of comorbidities is concerned. It is also possible that patients were not aware of their comorbidities as they had not been diagnosed before entering the country. Insufficient data collection on travel history, especially during the third wave of the outbreak in Vojvodina, when the health system was overwhelmed, could have resulted in underreporting of the travel-associated cases, and their participation in the total number of registered cases could be much higher than 0.83% reported here. Thirdly, we were unable to collect information on other lifestyle risk factors (smoking, diet, alcohol, etc.) and disease outcomes of travel-associated cases. Also, we lacked information about the length of stay abroad, reasons for travel, as well as the data about intensity of SARS-CoV-2 transmission in the countries from which the patient comes.

In conclusion, during the 2020, the SARS-CoV-2 virus was mainly imported into Vojvodina from neighboring countries, as well as from Austria and Germany. The results of our study emphasize that COVID-19 mainly affected the working-age population and males among travel-associated cases. We also found that the disease was most often manifested as mild, while severe and critical forms of the disease were more common in people with comorbidities and the elderly, which are recognized as a group with high risk for hospitalization. Also, our results underline that the predictors of pneumonia were: increasing age, male sex, presence as well as an increasing number of underlying comorbidities, an increasing number of days from the return to laboratory confirmation of COVID-19 and an increasing number of days from symptoms onset to diagnosis. Additionally, experiencing general infectious or respiratory symptoms was also the predictor of pneumonia, while loss of smell and taste were protective factors for developing it. Travel-associated COVID-19 cases with obesity, diabetes or hypertension had the higher risk for developing more severe forms of COVID-19, than those with other reported comorbidities.

Based on the consistency of our findings with the results of other studies, which analyzed all COVID-19 cases in the general population, we can assume that the epidemiological and clinical characteristics of travel-associated cases and cases of COVID-19 in the general population of Vojvodina do not differ significantly. Hence, our results at least approximately represent the epidemiological and clinical dynamics of COVID-19 disease in Vojvodina. Certainly, additional studies are needed to confirm or rule out this hypothesis.

## Supporting information

S1 Fig Time from entering the country until the onset of symptoms/signs in COVID-19 travel-associated cases in Vojvodina, Serbia, in the period March 6—December 31, 2020. (TIF)

S2 Fig Clinical presentation of COVID-19 travel-associated cases in Vojvodina, Serbia, in the period March 6—December 31, 2020, by sex. (TIF)

S1 Table Countries of importation of the SARS-CoV-2 to Vojvodina, Serbia, in the period March 6—December 31, 2020. (TIF)

S2 Table Study’s minimal underlying data set. (XLS)
