# Supplementary figures and images for: Epidemiological characteristics of COVID-19 travel-associated cases in Vojvodina, Serbia, during 2020
Source: PLoS One. 2021 Dec 23;16(12):e0261840. doi: 10.1371/journal.pone.0261840 (PMC8699980; doi:10.1371/journal.pone.0261840)

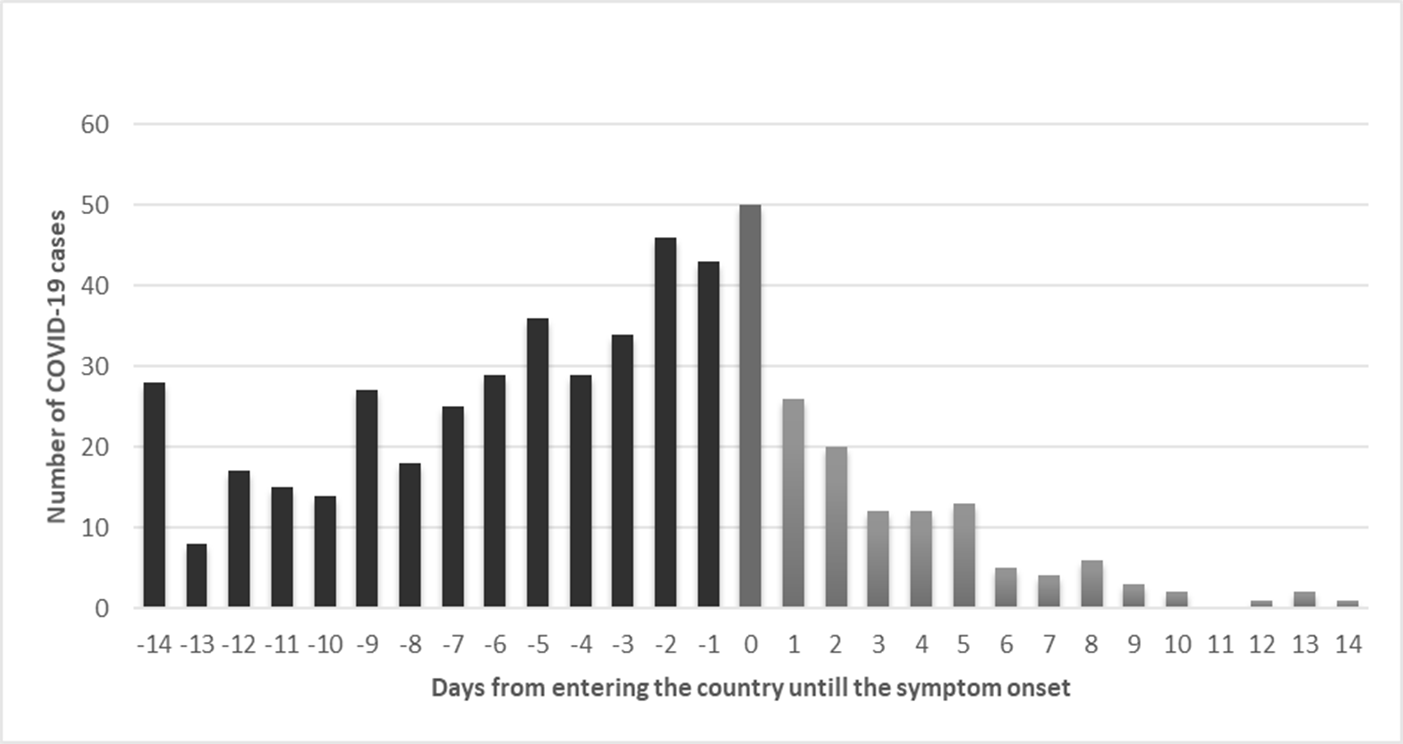

Supplement: S1 Fig — (TIF) [file pone.0261840.s001.tif]

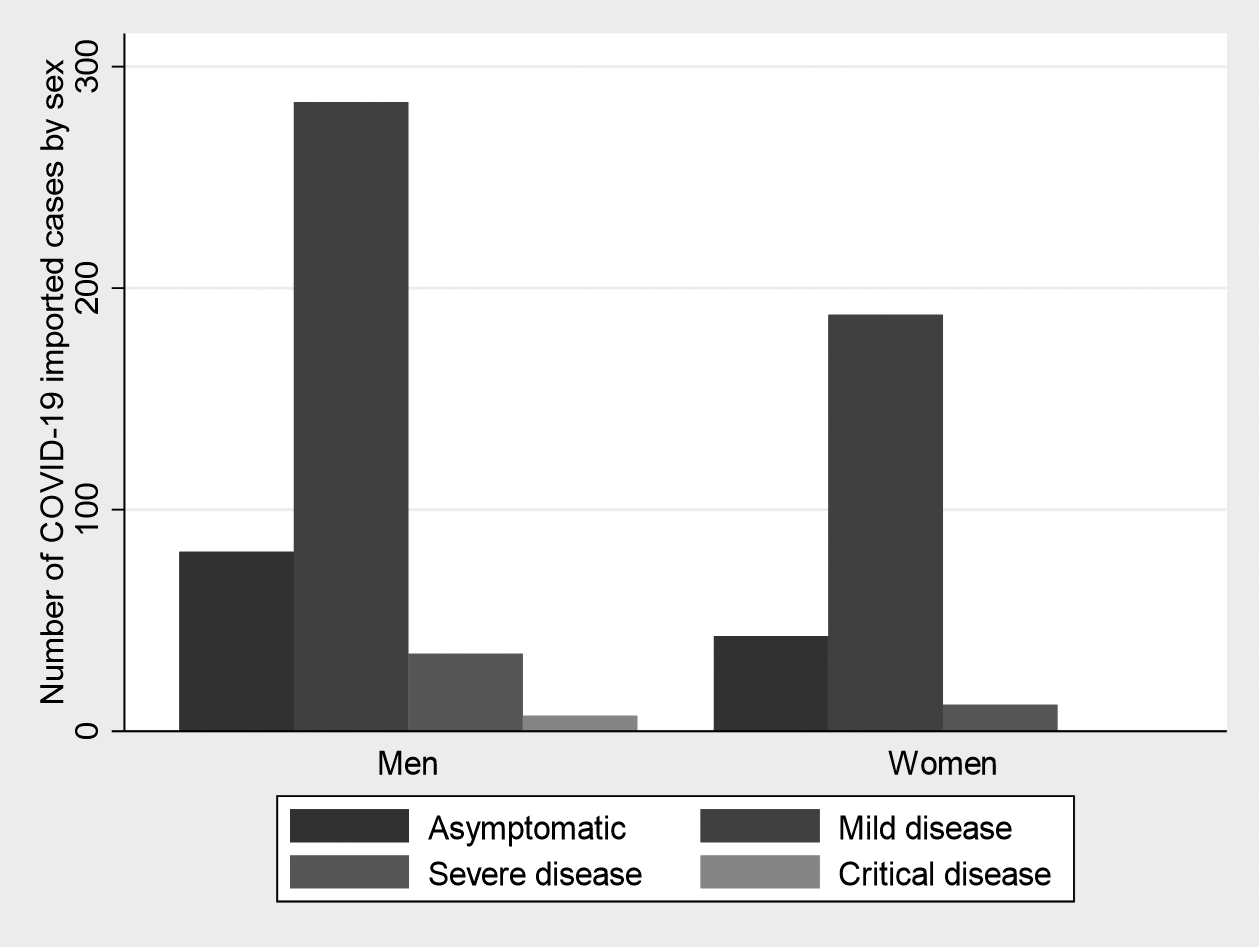

Supplement: S2 Fig — (TIF) [file pone.0261840.s002.tif]

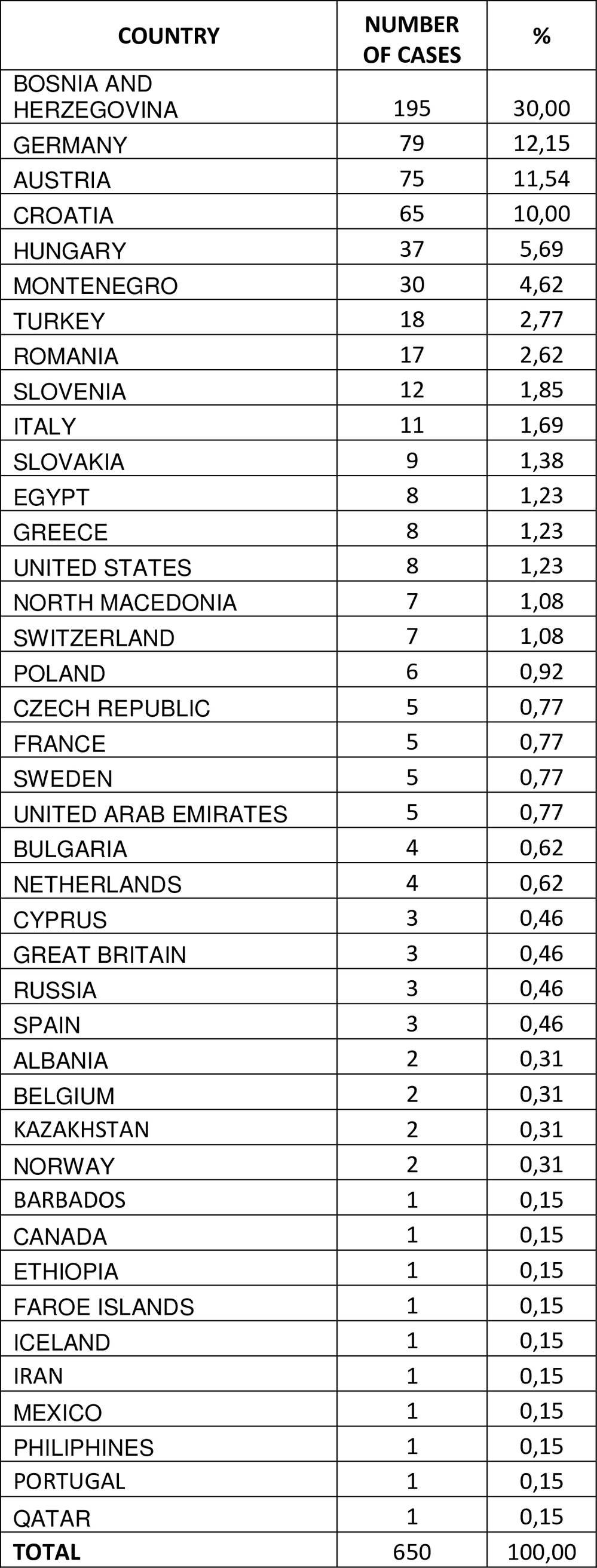

Supplement: S1 Table — (TIF) [file pone.0261840.s003.tif]
